# Loneliness increases the risk of type 2 diabetes: a 20 year follow-up – results from the HUNT study

**DOI:** 10.1007/s00125-022-05791-6

**Published:** 2022-09-28

**Authors:** Roger E. Henriksen, Roy M. Nilsen, Ragnhild B. Strandberg

**Affiliations:** Faculty of Health and Social Sciences, Western Norway University of Applied Sciences, Bergen, Norway

**Keywords:** Depression, HUNT study, Insomnia, Loneliness, Type 2 diabetes

## Abstract

**Aims/hypothesis:**

Type 2 diabetes is one of the leading causes of death globally and its incidence has increased dramatically over the last two decades. Recent research suggests that loneliness is a possible risk factor for type 2 diabetes. This 20 year follow-up study examined whether loneliness is associated with an increased risk of type 2 diabetes. As both loneliness and type 2 diabetes have been linked to depression and sleep problems, we also investigated whether any association between loneliness and type 2 diabetes is mediated by symptoms of depression and insomnia.

**Methods:**

We used data from the Trøndelag Health Study (HUNT study), a large longitudinal health study based on a population from central Norway (*n*=24,024). Self-reports of loneliness (HUNT2 survey, 1995–1997) and data on HbA_1c_ levels (HUNT4 survey, 2017–2019) were analysed to evaluate the associations between loneliness and incidence of type 2 diabetes. Associations were reported as ORs with 95% CIs, adjusted for sex, age and education. We further investigated the role of depression and insomnia as potential mediating factors.

**Results:**

During the 20 year follow-up period, 4.9% of the study participants developed type 2 diabetes. Various degrees of feeling lonely were reported by 12.6% of the participants. Individuals who felt most lonely had a twofold higher risk of developing type 2 diabetes relative to those who did not feel lonely (adjusted OR 2.19 [95% CI 1.16, 4.15]). The effect of loneliness on type 2 diabetes was weakly mediated by one subtype of insomnia but not by symptoms of depression.

**Conclusions/interpretation:**

This study suggests that loneliness may be one factor that increases the risk of type 2 diabetes; however, there is no strong support that the effect of loneliness on type 2 diabetes is mediated by depression or insomnia. We recommend that loneliness should be included in clinical guidelines on consultations and interventions related to type 2 diabetes.

**Graphical abstract:**

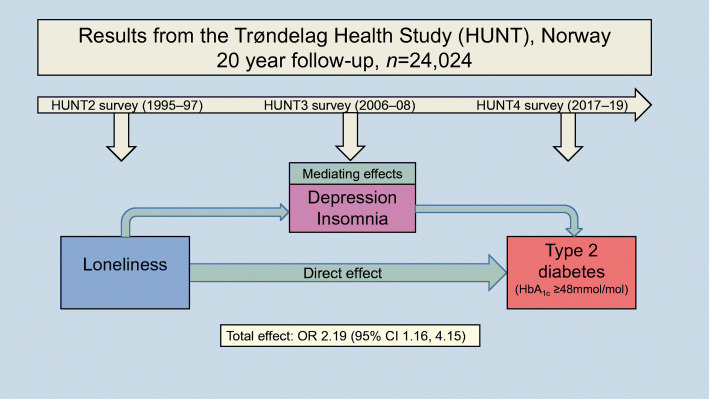

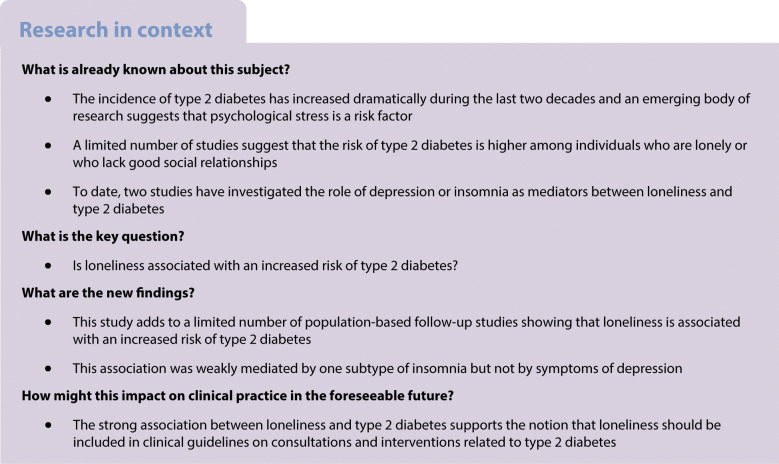



## Introduction

Loneliness is a painful feeling reflecting a state of distress linked to a perceived mismatch between the quantity and quality of the social relationships that we have and the quantity and quality of those that we want [1]. A growing body of literature suggests that there is a link between psychological stress and type 2 diabetes [2]. In line with previous studies on associations between loneliness and type 2 diabetes, the present study builds on the assumption that loneliness represents a state of psychological stress that may cause a general activation of the body’s stress responses, leading to an increased risk of type 2 diabetes [3–5]. Although the exact mechanisms are not fully understood, it is believed that activation of physiological stress responses over time plays a central role in the aetiology of type 2 diabetes [6]. This involves responses of the adrenergic nervous system and the hypothalamic–pituitary–adrenal (HPA) axis [1]. For instance, HPA axis activation leads to elevated secretion of cortisol, which in turn leads to increased glycogenolysis and temporary insulin resistance. Notably, this process also involves regulation by the brain of appetite and eating behaviour, including an increase in appetite for carbohydrates, with subsequent elevated blood sugar levels [7–9]. For example, it has been demonstrated that higher scores on loneliness are associated with higher consumption of sugary beverages and foods rich in sugars and fats [7, 9]. Against this background, given that loneliness induces a state of chronic stress and may lead to unhealthy eating behaviour, it is reasonable to assume that there is a direct link between loneliness and the risk of developing type 2 diabetes.

Research that aims to establish whether loneliness is a risk factor for the development of type 2 diabetes is still in its early days. However, the few studies that do exist indicate that the association between loneliness and type 2 diabetes may be of clinical importance. In a 5 year follow-up study (*n*=24,687), Christiansen et al [5] found that loneliness increased the risk of type 2 diabetes, with an HR of 1.98 for lonely participants compared with non-lonely participants. Similarly, in a longitudinal study carried out from 2004 to 2017 (*n*=4112), loneliness was found to be a significant predictor of type 2 diabetes, with an HR of 1.46 [3]. Positive associations between loneliness and type 2 diabetes have also been reported in cross-sectional studies [4, 10, 11]. An exception is the study by Pengpid and Peltzer [12], who reported a statistically non-significant association between loneliness and self-reported high blood sugar or diabetes. In that study, however, no distinction was made between type 1 and type 2 diabetes.

Insomnia, sleep deprivation and interrupted sleep have been found to be related to an increased risk of type 2 diabetes [13–15]. Studies have also shown that poorer sleep efficiency and more time awake after sleep onset are more frequently seen in lonely individuals than in non-lonely individuals, and it has been suggested that higher vigilance and higher activation of stress responses among lonely individuals may contribute to such sleep problems [16, 17]. To our knowledge only two previous studies (based on the same survey) have found that the association between loneliness and diabetes was mediated by sleep [5, 11]. Several indicators of sleep quality have been associated with loneliness [18]. In the adult Norwegian population, insomnia is the most prevalent sleep disorder [19]. We therefore included insomnia as a possible mediator between loneliness and type 2 diabetes in our study.

It has been established that depressive symptoms are associated with a significantly increased risk of type 2 diabetes [20–22]. It has also been reported that loneliness can lead to depression [16, 23]. In terms of biological responses, loneliness and depression have some shared pathways, affecting the HPA axis, the immunoinflammatory system and the regulation of energy metabolism, which mechanistically could explain a link to type 2 diabetes [1, 2]. However, to date there is little evidence that depression acts as a mediator between loneliness and type 2 diabetes. Recently, Christiansen et al [5] reported that ‘negative affect’, a two-item variable that included self-reported statements of both anxiety and depression, mediated the relationship between loneliness and type 2 diabetes. Hence, in the present study we included depression as a second possible mediator.

**Fig. 1:**
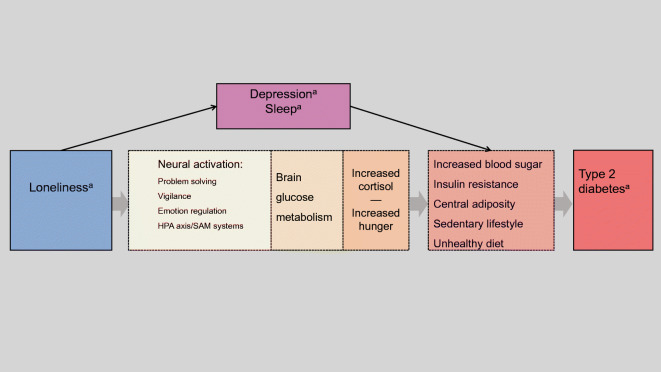
Model illustrating how physiological stress responses may be activated by loneliness (or by the absence of supporting relationships). Importantly, the sustained activation of physiological stress responses leads to changes in the cardiovascular system and cortisol production. This in turn may lead to increased food intake, in particular the intake of carbohydrates, and to increased insulin resistance. These processes play an important role in supplying the activated, metabolically demanding brain with sufficient glucose. Loneliness may also lead to depressive symptoms and/or sleep disturbances, which alter cortisol and glucose levels and increase the risk of type 2 diabetes. ^a^The following associations were tested: loneliness (exposure), depression and sleep (mediators) and type 2 diabetes (outcome). SAM, sympathetic adrenal medullary

A theoretical model showing the study variables in relation to each other is outlined in Fig. 1. The model includes potential mechanisms that may link loneliness to type 2 diabetes (e.g. increased neural activation, increased brain glucose metabolism, altered levels of cortisol, insulin resistance, increased hunger and a sedentary lifestyle). It should be noted that the present study was not designed to investigate such potential mechanisms or to investigate possible bidirectional relationships between the study variables, but to place the study variables in a frame of previous research demonstrating that psychological stress can alter a range of bodily processes, which ultimately may lead to type 2 diabetes. For a more thorough elaboration of the rationale and the research that supports our theoretical model, see Henriksen [24].

The present study was designed to examine the effect of loneliness on type 2 diabetes, based on a broad population sample (the Trøndelag Health Study [HUNT Study]) and a 20 year follow-up period. The possible mediating effects of insomnia and depression were also examined.

## Methods

### Participants

We used data from the HUNT study, which is a collaboration between the HUNT Research Centre (Faculty of Medicine and Health Sciences, Norwegian University of Science and Technology [NTNU]), Trøndelag County Council, the Central Norway Regional Health Authority and the Norwegian Institute of Public Health. In total, more than 230,000 people have consented for their health information (based on self-report questionnaires, medical examinations and blood samples) to be made available for research. Data were obtained through four population surveys: HUNT1 (1984–1986), HUNT2 (1995–1997), HUNT3 (2006–2008) and HUNT4 (2017–2019). In the present study, data from the HUNT2, HUNT3 and HUNT4 surveys were used. In the HUNT2 survey, 65,228 individuals aged 20 years or older agreed to participate (69.5% of invitees). In the HUNT3 and HUNT4 surveys, 50,800 (54.1% of invitees) and 56,042 (54.0% of invitees) individuals, respectively, agreed to participate. A total of 34,992 participants provided data for all three surveys. The HUNT2–4 surveys are described in more detail elsewhere [25].

### Exclusion criteria

Participants with self-reported type 1 diabetes or who met the criteria for type 2 diabetes (based on blood samples) and those who had a metabolic disorder (measured as self-reported hypothyroidism, hyperthyroidism or use of medications for metabolic disorders) at baseline were excluded from our analyses (*n*=1871). An additional 9097 participants were excluded because of missing data on HbA_1c_ in the HUNT4 survey. Following these exclusions, data provided by 24,024 participants were included in our analyses.

### Type 2 diabetes (outcome)

Type 2 diabetes status was the main outcome variable and was derived from blood sample analysis of HbA_1c_ recorded in the HUNT4 survey (2017–2019). The clinical criterion for diagnosis of type 2 diabetes was HbA_1c_ ≥48mmol/mol (≥6.5%) [26]. Further details about the data collection procedures used are available in Krokstad et al [27].

### Loneliness (exposure)

The exposure variable, loneliness, was measured by asking participants the following question: ‘In the last 2 weeks, have you felt lonely?’. Responses were given on a four-point scale (‘no’, ‘a little’, ‘a good amount’ and ‘very much’). Data for the exposure variable were obtained from the HUNT2 survey (1995–1997). Measuring loneliness using a single item has been found to correlate highly with measurement using larger scales, such as the UCLA Loneliness Scale (ULS-8) [28].

### Hospital Anxiety and Depression Scale (mediator)

To measure symptoms of depression we used the Hospital Anxiety and Depression Scale (HADS). The full version of HADS consists of 14 questions: seven questions related to anxiety and seven questions related to depression [29]. For our purpose, we used only the data from the depression subscale (HADS-D), derived from the HUNT3 survey (2006–2008). Each question was scored on a scale of 0–3; the total score ranged from 0 to 21, with higher scores indicating higher levels of depressive symptoms.

### Insomnia (mediator)

To measure insomnia, we used the HUNT sleep questionnaire included in the HUNT3 survey [30]. The instrument consists of nine items about snoring, sleep apnoea, sleepiness, restless legs, morning headache, night sweating and three different types of insomnia. For the purpose of the present study, we included the items on sleep-onset insomnia, sleep maintenance insomnia and terminal insomnia: ‘How often in the last 3 months have you …’ ‘had difficulty falling asleep at night’, ‘woken up repeatedly during the night’ and ‘woken too early and couldn’t get back to sleep’, respectively. In the HUNT3 survey questionnaire there are three response options for each question (‘never/seldom’, ‘sometimes’ and ‘several times a week’).

### Control variables

Analyses were adjusted for the following socioeconomic factors: age (years at baseline), sex (male, female) and education (highest level obtained among four categories: elementary school, non-university lower education, university <4 years, university at least 4 years). Because of the non-linear relation between age and type 2 diabetes, age was included as a quadratic term (i.e. age + age^2^) in regression models to obtain better confounding adjustment for the associations between loneliness and type 2 diabetes. All adjustment factors were selected a priori by subject matter knowledge and evaluated as confounding factors using directed acyclic graphs [31].

### Statistical analyses

All analyses were performed in Stata release 16.1 (StataCorp LLC, College Station, TX, USA) for Windows.

To examine the association between self-reported loneliness and type 2 diabetes, we performed binary logistic regression analyses using the exposure category ‘No, I have not felt lonely’ as the reference group. The associations were reported by crude and adjusted ORs with 95% CIs; the adjusted ORs were adjusted for sex, age (as a continuous quadratic term) and education. In all regression analyses, missing data in model variables were handled using a multiple imputation algorithm. This was done by creating 100 imputed datasets using the fully conditional specification and sequential chained equations as implemented in the ‘mi’ suite of commands in Stata [32]. The imputation model included all variables contained in the logistic regression models and mediation analyses (see below), and the pooling of ORs with 95% CIs across imputed datasets was performed using Rubin’s combination rules [33].

To examine if self-reported depression or insomnia mediated the total effect of loneliness on type 2 diabetes, we performed a counterfactual-based mediation analysis using the ‘ldecomp’ command in Stata [34, 35]. This approach allowed us to decompose the OR total effect for the association between loneliness and type 2 diabetes into the OR direct effect (the effect of loneliness on type 2 diabetes through pathways that do not involve the mediator) and the OR indirect effect (the effect of loneliness on type 2 diabetes caused by the effect of loneliness on the mediator). The ‘ldecomp’ command further allowed for the inclusion of exposure-by-mediator interactions as well as adjustments for confounding factors. Finally, multiple mediators are also allowed, and these can follow any probability distribution.

In the present analysis, we performed separate mediation analyses for depression (as a continuous variable) and the three types of insomnia (as categorical variables) and hypothesised that the effects of depression and insomnia on type 2 diabetes would vary according to the level of loneliness (i.e. loneliness-by-mediator interaction). The total, direct and indirect effects were reported using ORs, adjusted for participants’ sex, age (as a continuous quadratic term) and education. Uncertainty in all effect estimates was obtained using bootstrap CIs, which were based on 2000 replications. The mediation analyses were performed on the previous 100 imputed datasets using the chained equations algorithm. The main requirement for mediation was that the indirect effect was statistically significant [36].

All results reported in the text and tables (except Table 1) are based on multiply imputed data only. Results based on complete case analysis showed essentially the same effects, except for the highest category of loneliness, ‘very much’, which appeared stronger in logistic regression analyses and mediation analyses (data not shown).

**Table 1:** Characteristics of the participants who completed the HUNT2 (1995–1997), HUNT3 (2006–2008) and HUNT4 (2017–2019) surveys by type 2 diabetes status

Characteristic	Type 2 diabetes^a^		*p* value^b^
	No (*n*=22,845)	Yes (*n*=1179)	
Sex^c^			<0.001
Female	12,785 (56.0)	480 (40.7)	
Male	10,060 (44.0)	699 (59.3)	
Age at screening (years), mean (SD)^c^	43.3 (11.2)	47.9 (9.8)	<0.001
Age at screening (years), median (IQR)^c^	43.4 (34.7, 51.3)	48.4 (41.3, 54.5)	<0.001
HbA_1c_ (mmol/mol), mean (SD)^d^	34.9 (3.6)	57.4 (10.5)	<0.001
HbA_1c_ (%), mean (SD)^d^	5.3 (0.3)	7.4 (1.0)	<0.001
Marital status^c^			<0.001
Unmarried	5456 (23.9)	184 (15.6)	
Married	15,422 (67.5)	862 (73.1)	
Divorced/widow/widower	1921 (8.4)	132 (11.2)	
Missing	46 (0.2)	1 (0.1)	
Education^c^			<0.001
Elementary school	5334 (23.3)	410 (34.8)	
Non-university lower education	11,357 (49.7)	551 (46.7)	
University <4 years	3603 (15.8)	132 (11.2)	
University at least 4 years	2297 (10.1)	64 (5.4)	
Missing	254 (1.1)	22 (1.9)	
In the last 2 weeks, have you felt lonely?^c^			0.039
No	18,743 (82.0)	931 (79.0)	
A little	2337 (10.2)	127 (10.8)	
A good amount	423 (1.9)	24 (2.0)	
Very much	95 (0.4)	11 (0.9)	
Missing	1247 (5.5)	86 (7.3)	
HADS-D score, mean (SD)^e^	3.2 (2.8)	3.6 (2.9)	<0.001
Sleep-onset insomnia^e^			0.34
Never/seldom	9194 (40.2)	457 (38.8)	
Sometimes	8074 (35.3)	443 (37.6)	
Several times a week	2045 (9.0)	109 (9.2)	
Missing	3532 (15.5)	170 (14.4)	
Sleep maintenance insomnia^e^			<0.001
Never/seldom	6060 (26.5)	255 (21.6)	
Sometimes	9571 (41.9)	537 (45.5)	
Several times a week	3659 (16.0)	213 (18.1)	
Missing	3555 (15.6)	174 (14.8)	
Terminal insomnia^e^			0.18
Never/ seldom	8937 (39.1)	436 (37.0)	
Sometimes	8187 (35.8)	451 (38.3)	
Several times a week	2140 (9.4)	118 (10.0)	
Missing	3581 (15.7)	174 (14.8)	

Data are *n* (%) unless indicated otherwise

^a^HUNT4 survey (2017–2019). Diabetes ‘no’: HbA_1c_ <48 mmol/mol (<6.5%); diabetes ‘yes’: HbA_1c_ ≥48 mmol/mol (HbA_1c_ ≥6.5%)

^b^Two-sided *p* values were obtained using the two-sample *t* test for continuous data and the χ^2^ test for categorical data

^c^HUNT2 survey (1995–1997)

^d^HUNT4 survey (2017–2019)

^e^HUNT3 survey (2006–2008)

### Ethics

The study was approved by the Regional Committee for Medical and Health Research Ethics (reference number 2017/45) and was considered to represent a minor ethical challenge. Caution should be taken when presenting the results as some people diagnosed with type 2 diabetes may feel stigmatised if this is associated with loneliness.

## Results

Table 1 shows the characteristics of the study sample by type 2 diabetes diagnosis. Among the 24,024 participants included in our analyses, 1179 (4.9%) developed type 2 diabetes during the study period (1995–2019). Individuals with type 2 diabetes were more often men (59.3%) and had a higher mean age (47.9 years vs 43.3 years) than those without type 2 diabetes. Moreover, individuals with type 2 diabetes reported more frequently than individuals without type 2 diabetes that they were married (73.1% vs 67.5%) and that they had the lowest education level (34.8% vs 23.3%). Among the participants included in the study, various degrees of feeling lonely were reported by 12.6%.

Results from the logistic regression analyses (Table 2) showed that study participants reporting higher levels of loneliness were associated with higher ORs for type 2 diabetes (per category change in loneliness: adjusted OR 1.13 [95% CI 1.00, 1.28]; *p*_trend_=0.047). Specifically, participants who responded ‘very much’ to the question on experiencing loneliness during the last 2 weeks in the HUNT2 survey (1995–1997) had two times higher odds for type 2 diabetes 20 years later in the HUNT4 survey (2017–2019) relative to those who had not felt lonely (adjusted OR 2.19 [95% CI 1.16, 4.15]).

**Table 2:** Logistic regression analyses for the association between loneliness and type 2 diabetes: HUNT surveys (1995–2019)

In the last 2 weeks, have you felt lonely?	No. of subjects	No. (%) with type 2 diabetes	Crude OR (95% CI)^a^	Adjusted OR (95% CI)^b^
No^c^	20,800	1002 (4.8)	1.00	1.00
A little	2633	139 (5.3)	1.10 (0.91, 1.33)	1.12 (0.92, 1.36)
A good amount	478	26 (5.4)	1.15 (0.76, 1.74)	1.08 (0.71, 1.63)
Very much	113	12 (10.6)	2.22 (1.19, 4.16)	2.19 (1.16, 4.15)
Per category change^d^			1.14 (1.01, 1.29) [0.032]^e^	1.13 (1.00, 1.28) [0.047]^e^

^a^Estimated from the logistic regression model

^b^Adjusted for sex, age (as a quadratic term) and education

^c^Reference group

^d^By incorporating loneliness as a linear term in the logistic regression model

^e^*p* value for trend across loneliness categories

Results from the mediation analyses (Table 3) showed that the total effect of self-reported loneliness on type 2 diabetes was not mediated by symptoms of depression after adjusting for participants’ sex, age and education (the 95% CIs for all OR indirect effects overlapped 1.00). We also did not find that the effect was mediated by sleep-onset insomnia or by terminal insomnia (data not shown). However, when examining sleep maintenance insomnia as a mediator (Table 3), the 95% CIs for the adjusted OR indirect effects for the loneliness groups ‘a little’ and ‘a good amount’ did not entirely overlap 1.00, suggesting mediating effects of sleep maintenance insomnia.

**Table 3:** Mediation analysis of the association between loneliness and type 2 diabetes by symptoms of depression or insomnia: HUNT surveys (1995–2019)

In the last 2 weeks, have you felt lonely?	Adjusted OR (95% CI)^a^		
	Total effect	Indirect effect	Direct effect
Mediation analyses for depression			
No^b^	1.00	1.00	1.00
A little	1.12 (0.93, 1.36)	1.02 (0.98, 1.06)	1.10 (0.91, 1.34)
A good amount	1.08 (0.70, 1.66)	1.03 (0.97, 1.10)	1.05 (0.68, 1.61)
Very much	2.29 (1.12, 4.66)	1.04 (0.96, 1.12)	2.20 (1.07, 4.50)
Mediation analyses for sleep maintenance insomnia			
No^b^	1.00	1.00	1.00
A little	1.12 (0.92, 1.36)	1.02 (1.00, 1.03)	1.10 (0.91, 1.34)
A good amount	1.10 (0.71, 1.68)	1.04 (1.00, 1.07)	1.06 (0.69, 1.63)
Very much	2.22 (1.10, 4.49)	1.03 (0.99, 1.06)	2.17 (1.07, 4.38)

^a^Adjusted for sex, age (as a quadratic term) and education. The model also included a loneliness-by-mediator interaction

^b^Reference group

## Discussion

We aimed to study to what degree loneliness is a risk factor for type 2 diabetes. We further examined if any association between loneliness and type 2 diabetes was mediated by insomnia and depression. Consistent with our main hypothesis we found that higher levels of loneliness at baseline were strongly associated with a higher risk of type 2 diabetes as measured 20 years later. We also found a statistically significant, but weak, mediating effect of sleep maintenance insomnia. However, the results did not support our hypotheses that the association between loneliness and type 2 diabetes is mediated by sleep-onset insomnia, terminal insomnia or symptoms of depression.

Our main finding confirmed the results from two recently published prospective studies on loneliness and the risk of type 2 diabetes [3, 5]. In one of the studies, based on a Danish health survey (*n*=24,687), Christiansen et al [5] found that, after adjusting for sex, age and education, loneliness increased the risk of type 2 diabetes compared with non-lonely participants, with an HR of 1.98. The other study, by Hackett et al [3], was based on a sample of individuals aged 50 and older living in England (*n*=4112). The authors found that loneliness was a significant predictor of type 2 diabetes, with an HR of 1.46 after adjusting for a range of variables such as demographic variables, alcohol consumption, physical activity level and BMI.

The current study is one of the first population-based studies to examine the association between loneliness and type 2 diabetes. The strengths of this study include the objectively measured HbA_1c_ levels, the large sample size and the prospective design, with self-reported data on loneliness collected long before HbA_1c_ data. After adjusting for age, sex and education level, we found that participants who responded ‘very much’ to the question on experiencing loneliness had two times higher odds of type 2 diabetes 20 years later than those who had not felt lonely (adjusted OR 2.19 [95% CI 1.16, 4.15]). Taking into consideration the differences in the study designs, there is little difference in the strength of the results between this study and the two above-mentioned studies [3, 5]. Our main result also agrees with the results from cross-sectional studies examining the associations between loneliness and type 2 diabetes [4, 10, 11] and the results from studies of factors related to loneliness, such as social isolation and quality of social relationships, and the incidence of type 2 diabetes [5, 37, 38]. However, the effects measured in our study should still be interpreted with some caution. First, we did not have complete follow-up data from the HUNT4 survey (HbA_1c_) for those who participated in the HUNT2 survey (loneliness). Further, we excluded a large number of participants who lacked data on HbA_1c_ in the HUNT4 survey (although missing data for essential covariates were multiply imputed). Loss to follow-up and the exclusion of participants because of missing outcome data are both potential sources of bias and could therefore have affected the effect measures to some extent in the present study [39].

The time between the surveys may also have affected the results. Findings from a meta-analysis based on 75 longitudinal studies imply that loneliness is a relatively stable phenomenon [40]. However, during a period of 10–20 years there is clearly much room for individual changes in loneliness, depressive symptoms and insomnia. Moreover, as the classification of type 2 diabetes was based on HbA_1c_ level and did not include medication use, it is also possible that some individuals with type 2 diabetes and glucose levels regulated by medications may have been classified as participants without type 2 diabetes. Another concern is that ‘loneliness’ was measured based on a single item. Although measuring loneliness with a single item has been found to correlate highly with loneliness measured using larger scales [28], this approach may still be regarded as a limitation, as a single-item measure does not distinguish between different types of loneliness (e.g. emotional and social loneliness). Another limitation is that most variables, including insomnia and depression, were measured using self-report questionnaires. A general concern of self-report questionnaires is that participants may provide inaccurate answers.

Different explanations have been provided for how loneliness may contribute to the pathogenesis of type 2 diabetes. As illustrated in Fig. 1, we built our main hypothesis on a theoretical model emphasising the physiological pathways linked to loneliness. The theoretical model is particularly supported by experimental laboratory studies of social isolation or stress. One example is the ‘hand-holding’ study by Coan et al [41], a functional magnetic resonance imaging (fMRI) study in which 16 women were confronted with the threat of mild electric shocks while alone, while holding a stranger’s hand or while holding their partner’s hand. The neural stress responses (such as amygdala activation) reached their highest levels when the women were exposed to the threat of electric shocks while alone, whereas they were at their lowest levels when the women were holding their partner’s hand [41]. The interpretation of the results was that isolated individuals require more neural metabolic resources in order to regulate their emotions [42]. Following this line of thinking, it is interesting to look at the work of Peters and colleagues [8], which explains how the brain’s energy supply is regulated through the stress system. In one of their experiments, it was demonstrated that social stress, induced by the Trier social stress test, was associated with increased levels of cortisol, a higher intake of carbohydrates and a suppressive effect on serum insulin, and ultimately influenced the levels of circulating glucose [8]. Recently, Pourriyahi et al [43] published a review on loneliness and its impact on immunological and metabolic illnesses and biomarkers that adds support to this type of psychoneuroimmunological explanation.

Although we clearly find the above-mentioned view plausible, our study was not aimed at examining such mechanisms directly, and several other mechanisms may be involved. Social support, social influence and social engagement may have positive effects on health-promoting behaviours. For example, appraisal or informational support from a friend may directly influence an individual’s health-related choices and subsequently have positive effects on physical activity, diet and BMI. Moreover, being socially engaged in, for example, sports clubs, religious groups or charity groups may influence an individual’s lifestyle through shared norms [44]. In contrast, as loneliness is associated with fewer social ties, the potential positive influences of such ties on lifestyle factors such as eating healthy food and exercising regularly will be lacking, making lonely individuals vulnerable to behaviour that could increase the risk of type 2 diabetes. This is another type of reasoning that also provides a plausible explanation for our results. However, behaviour- and psychoneuroimmunology-oriented explanations are not mutually exclusive; they may well both apply at the same time or even more likely have synergistic effects [7, 24].

Our mediation analysis showed a mediating effect of sleep maintenance insomnia that was statistically significant. This was in line with our expectations and theory that loneliness may lead to a general activation of stress responses and higher levels of vigilance [45]. High levels of stress hormones and activated neurotransmitters counteract sleep maintenance and increase the likelihood of frequent night waking [46]. Lack of sound sleep may lead to sustained high levels of stress hormones and insufficient nightly restorative processes, which may increase the risk of type 2 diabetes [11, 45]. Our finding harmonises with those of two different studies by Christiansen et al [5, 11], who found that self-reported poor sleep quality and poor sleep duration are significant mediators in the association between self-reported loneliness and diabetes. In our study, the mediating effects of sleep-onset insomnia and terminal insomnia were not statistically significant. The insomnia subtypes may derive from dysregulation of different processes involved in sleep homeostasis [46, 47], and it is possible that the mechanisms involved in sleep maintenance insomnia and the loneliness–type 2 diabetes relationship are somewhat different from the mechanisms involved in sleep-onset insomnia and terminal insomnia. It should, however, be noted that the mediating effect of sleep maintenance insomnia was weak and this result should therefore be interpreted with caution.

Contrary to our expectations, we did not find support for the hypothesis that depressive symptoms mediate the association between loneliness and type 2 diabetes. This was unexpected as loneliness has previously been recognised as a major risk factor for depression [16, 23], while depression has been reported to be a risk factor for type 2 diabetes [20, 21]. The mediation results contradict the results of a study by Christiansen et al [5], who found that symptoms of anxiety and depression did mediate the association between loneliness and type 2 diabetes. Despite the strengths of our study, with its large cohort and long follow-up, we therefore cannot reject the hypothesis that the association between loneliness and type 2 diabetes is mediated by depressive symptoms. In fact, the long follow-up time and timing of the assessments in our study may have led to limitations when it comes to studying the mediating effects of depression on type 2 diabetes. Depression was reported 10 years after the main exposure variable (loneliness) and 10 years before the outcome variable (type 2 diabetes). While loneliness is a relatively stable phenomenon, especially when linked to personality traits, depression is often episodic and may last for shorter or longer periods, with large variability in severity and duration both within and between individuals [40]. A similar methodological concern could also be raised regarding insomnia. Studies with higher frequencies of assessments may be more suitable to study depression and insomnia as mediating factors between loneliness and type 2 diabetes.

In conclusion, in this 20 year follow-up study we found that a high degree of loneliness was associated with a twofold risk of type 2 diabetes. In our study, this association was not mediated by depression and was mediated to only a very small degree by sleep maintenance insomnia. We recommend that loneliness should be included in clinical guidelines on consultations and interventions related to type 2 diabetes [48]. It is important that healthcare providers are open to dialogue about an individual’s concerns during clinical consultations, including with regard to loneliness and social interaction. In an experimental study of older people living in a senior citizen apartment building, Arnetz et al [49] demonstrated that participation in a 6 month social activation programme was associated with reduced levels of HbA_1c_. This result is encouraging with regard to future studies aimed at investigating other potential preventive interventions for type 2 diabetes.

We recommend that further research is carried out into the role of insomnia and depression in the relationship between loneliness and type 2 diabetes. The role of other factors such as diet, obesity and physical activity should also be investigated. These are well-known risk factors for type 2 diabetes and have also been associated with loneliness or social isolation [10]. More research on the direct link between loneliness and type 2 diabetes is also needed to better understand the mechanisms at play. Questions to be answered are the extent to which loneliness leads to the activation of stress responses, the extent to which loneliness affects health-related behaviour and, importantly, how these two pathways interact in terms of contributing to an increased risk of type 2 diabetes. Finally, it will be important to determine to what degree other constructs related to loneliness are associated with type 2 diabetes. Examples of such constructs are social isolation and personality styles such as shyness, avoidant attachment style and type D personality. We consider that the answers to these questions will be useful when planning targeted preventive interventions for type 2 diabetes.

## Data Availability

The HUNT study invited people aged 13–100 years to participate in four surveys between 1984 and 2019. Comprehensive data from more than 230,000 people who participated at least once and biological material from 120,000 people have been collected. The data are stored in the HUNT databank and biological material are stored in the HUNT biobank. The HUNT Research Centre has permission from the Norwegian Data Inspectorate to store and handle these data. The key identifier in the database is the personal identification number given to all Norwegians at birth or on immigration; de-identified data are sent to researchers on approval of a research protocol by the regional ethics committee and HUNT Research Centre. To protect participants’ privacy, the HUNT Research Centre aims to limit the storage of data outside the HUNT databank and cannot deposit data in open repositories. The HUNT databank has precise information on all data exported to different projects and can reproduce these data on request. There are no restrictions regarding data export given approval of applications to the HUNT Research Centre. For more information see https://www.ntnu.edu/hunt/data.

## Abbreviations

HADS: Hospital Anxiety and Depression Scale
HADS-D: Hospital Anxiety and Depression Scale, depression subscale
HPA: Hypothalamic–pituitary–adrenal
HUNT: Trøndelag Health Study
